# Challenging Reported Frizzled-Targeting Compounds in Selective Assays Reveals Lack of Functional Inhibition and Claimed Profiles

**DOI:** 10.1021/acsptsci.4c00570

**Published:** 2024-12-02

**Authors:** Alexey Koval, Cédric Boudou, Vladimir L. Katanaev

**Affiliations:** Department of Cell Physiology and Metabolism, Translational Research Centre in Oncohaematology, Faculty of Medicine, University of Geneva,1206 Geneva, Switzerland

**Keywords:** FZD antagonists, Wnt signaling, GPCR drug discovery, preclinical drug development

## Abstract

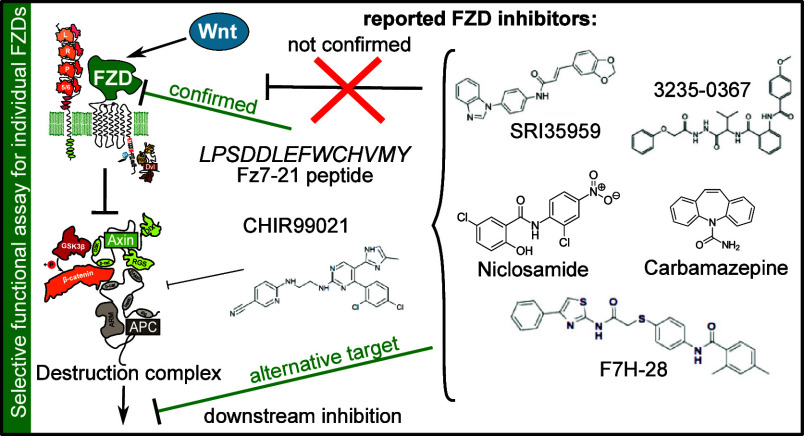

Selective inhibitors of Frizzled (FZD) GPCRs are highly sought after as potentially highly efficacious and safe treatments for cancer as well as tools in regenerative medicine and fundamental science. In recent years, there have been several reports claiming the identification of small molecule agents that are selective toward certain FZD proteins using a variety of approaches. However, the majority of these studies lacked a selective functional assay to validate their functionality. In this study, we describe the development and application of a selective assay for individual FZD proteins. Our findings indicate that the majority of reported compounds lack the capacity to inhibit the functioning of the claimed FZD proteins when stimulated by a Wnt ligand in the canonical pathway. Instead, the compounds demonstrate a broad range of off-target effects, including inhibition of downstream pathway component(s) (3235-0367, SRI35959, carbamazepine, niclosamide), lack of activity (FzM1), and surprising antagonism of firefly luciferase (F7H). The only compound that fulfills the expected selectivity profile is peptide Fz7–21. These results highlight the necessity of implementing rigorous testing of the screening-derived compounds in selective functional assays and are important for the field of drug discovery and development targeting the highly demanded Wnt-FZD pathway.

Oncology research has historically been one of the most productive areas of the pharmaceutical industry.^1^ However, this accelerated pace implies that only the most selective, safe, and targeted treatments can be expected to gain the regulatory approval, with marketed drugs targeting nearly all known cancer-associated signaling pathways.^1,2^ The Wnt signaling pathway, which was discovered nearly four decades ago, has emerged as a central player in carcinogenesis. Yet, it remains one of the few cancer-associated pathways for which no approved drugs are available.^3,4^

The pivotal function of aberrant Wnt signaling in cancer has driven the development of first-generation pharmacological agents that target this pathway. However, a significant challenge emerged: These drugs often cause on-target side effects due to their nonselective inhibition of Wnt signaling throughout the body.^3,5,6^ Wnt signaling plays a pivotal role in maintaining adult tissue homeostasis by regulating processes such as tissue renewal, stem cell proliferation, cell migration, and differentiation.^7,8^ With over 100 protein components identified, the majority function across diverse healthy tissues, while only a smaller subset may exhibit tissue specificity.^9^ Consequently, the next generation of Wnt pathway inhibitors must achieve a crucial balance: targeting tumor-specific aspects of the pathway while minimizing disruption of essential physiological processes.

Genes encoding key pathway components are frequently subjected to mutation or dysregulation in the context of cancer. In the initial stages of research, the Wnt pathway was primarily associated with β-catenin-dependent signaling. However, our understanding has evolved to encompass a broader spectrum of signaling events initiated by Wnt ligands and Frizzled (FZD) receptors, including β-catenin-independent branches. In humans, 19 Wnt proteins activate signaling through 10 FZD cell surface G protein-coupled receptors (GPCRs), which form a separate GPCR class designated class F possessing structural and cell biology peculiarities distinguishing FZDs from other classes.^10,11^ Several Wnt coreceptors including LRP5/6, RYK, ROR1, and ROR2 further modulate the pathway outputs and are thought to enable context-dependent engagement of specific intracellular signaling cascades, including those considered noncanonical (RYK, ROR), synonymous with those not involving β-catenin-dependent transcription.^5,7^

In the context of Wnt signaling, multiple FZDs have been implicated in the initiation and progression of various cancers, including colorectal, breast, ovarian, gastric, endometrial, pancreatic ductal adenocarcinoma, and others.^3,4^ One promising approach to targeting cancers in a modern, selective, and safe manner involves the development of anti-FZD molecules that would antagonize cancer-related Wnt signaling while sparing physiological pathway activities in healthy tissues.^6,12^ Not surprisingly, numerous research groups have sought to identify FZD-targeting compounds with a broad range of applications (structures of such compounds used in the current study are shown in Figure 1). As FDA-approved compounds may provide an expedient pathway to clinical trials for other disease indications, many researchers employ this repurposing approach.^13,14^ From a screen of FDA-approved drugs to identify compounds that could interfere with FZD internalization, a key step in Wnt signaling, niclosamide, an antihelminthic drug, emerged as a potential FZD_1_-targeting compound.^15^ The interaction between carbamazepine, another FDA-approved compound, and FZD_8_ was evaluated by surface plasmon resonance and confirmed by the crystal structure. Functional assays revealed that this interaction suppresses Wnt/β-catenin reporter.^16^

**Figure 1:**
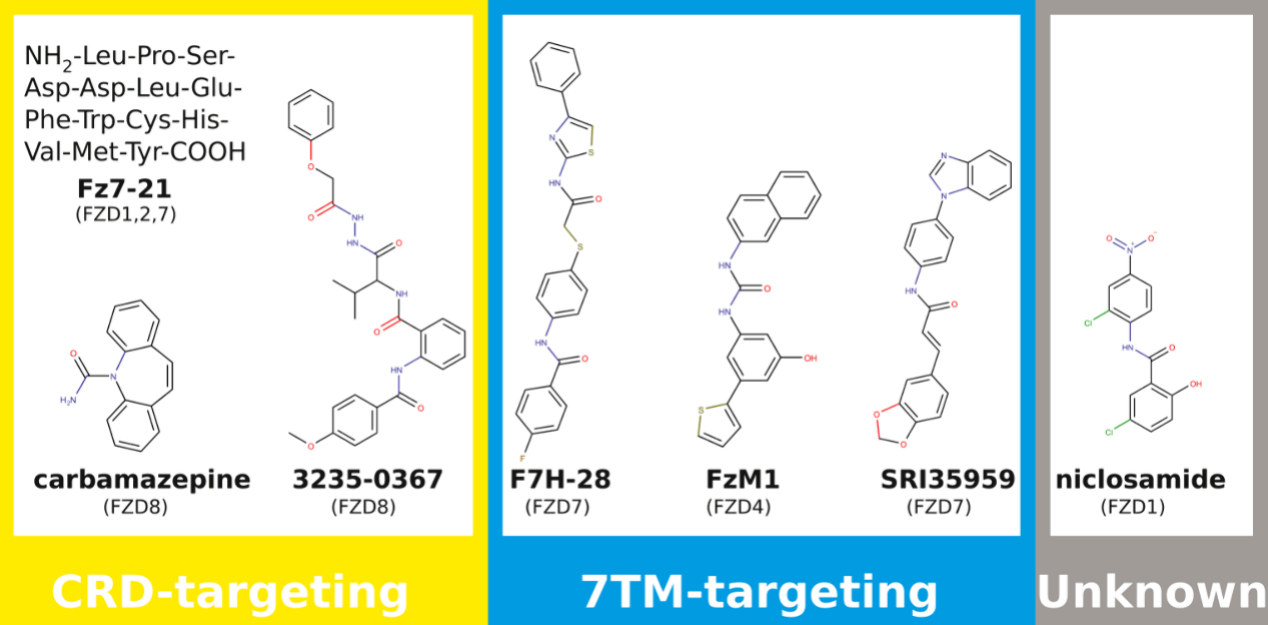
Compounds previously reported as selective FZD inhibitors, grouped by the part of the FZD they are designed to target (CRD = cysteine-rich domain, 7TM = 7-transmembrane domain) with indication of their claimed FZD targets below in brackets. Our study confirms only Fz7–21 as a functional FZD inhibitor.

The use of in silico-based approaches is becoming increasingly sophisticated, particularly with the advent of AI technologies, and is proving to be an effective method for significantly enhancing the yield of screening campaigns.^17^ A structure-based virtual screening approach was employed to identify small molecule inhibitors targeting the transmembrane domain (TMD) of FZDs. As a result, SRI37892 was demonstrated to be a potent inhibitor of Wnt signaling and cancer cell proliferation.^18^ In a separate study, another virtual screening-derived compound, 3235-0367, was assessed for its functional activity in 3T3 cells as a FZD_8_ CRD (extracellular N-terminal cysteine-rich domain)-targeting compound.^19^ In silico approaches that modeled the transmembrane domain of FZD_7_ resulted in the identification of compound F7H, which was found to inhibit the β-catenin-dependent Wnt signaling as measured by the transcriptional TopFlash assay in cells that had been stimulated in an autocrine manner by Wnt3a.^20^ In addition, through in silico approaches, a compound initially synthesized as a folding chaperone was identified as a negative allosteric modulator (NAM) of FZD_4_, renamed FzM1, and demonstrated to disrupt basal levels of the TopFlash reporter in FZD_4_-overexpressing HEK293 cells.^21^

The report of a peptide, Fz7–21, is a notable departure from the previous two lines of studies. This peptide was identified as selectively binding to CRD of several FZDs, namely, FZD_1,2, and 7_. Additionally, it was observed to disrupt Wnt signaling in cells endogenously expressing multiple FZDs.^22^

Although these studies purported to have identified potential FZD inhibitors, a common shortcoming was the lack of rigorous and selective functional validation, namely, measurements of the actual capacity of the compound to inhibit the target receptor in living cells. The conclusions about compounds’ selectivities were predominantly based on physical binding patterns or inferred from in silico data, rather than derived from direct evidence of the proposed target inhibition in living cells. In fact, the majority of cell lines standardly express multiple representatives of the 10-member FZD family, which complicates any analysis of FZD selectivity. For example, HEK293 cells report significant expression of FZD_1,3,4,6, and 7_ (with detectable levels of all others except FZD_10_),^23^ whereas in their HEK293T derivative, the dominant FZDs were FZD_2,3, and 7_ (with all others detectable except FZD_5 and 8_).^24^ Similarly, evaluation of the RNA-seq data set from 3T3 cells shows that they also express multiple FZDs, specifically FZD_1,2,7, and 8_, with all other homologues present at nonzero levels.^25^

Here, we utilized the recently developed HEK293T line with all 10 FZD proteins removed by sequential CRISPR-based knockout (ΔFZD_1–__10_ HEK293T cells)^26,27^; reintroduction of individual FZDs in this line permits us to build selective functional assays that overcome the major limitation of previous studies. Resultingly, we find that many reported compounds are in fact unable to inhibit the canonical Wnt signaling pathway in a FZD-dependent manner when stimulated by the Wnt ligand Wnt3a and cannot be regarded as FZD-selective agents. These results highlight the crucial need to use rigorous functional assays to assess the efficacy of screening-derived compounds as potential FZD inhibitors.

## Results

### ΔFZD_1__–__10_ HEK293T Cells Stably Transfected with the Firefly Luciferase-Based Transcriptional Wnt Reporter Provide a Reliable Platform to Test the Functional Activity of Individual FZDs upon Their Reexpression

The TopFlash Wnt reporter construct contains multiple TCF binding sites upstream of a minimal promoter controlling the firefly luciferase gene. Upon activation of the Wnt/β-catenin pathway, β-catenin translocates to the nucleus where it becomes a coactivator of the TCF/LEF transcription factors, allowing expression of luciferase and its intracellular accumulation. The resulting luminescence signal measured is robustly proportional to the activity of the Wnt/β-catenin pathway, providing an excellent functional readout. This assay has been a widely used workhorse in the Wnt signaling research, helping to identify and characterize various components and regulators of this pathway, as well as the functional consequences of genetic mutations or treatments affecting the Wnt/β-catenin pathway.^28,29^ It is also extremely useful in the screening and evaluation of various Wnt-targeting compounds. In such applications, it is often used in the context of a dual luciferase assay together with a non-homologous Renilla luciferase that is expressed independently from a constitutive promoter.^30−32^ In this manner, Renilla luciferase reports effects of the compounds under study on the general cell transcription (and well-being), thus differentiating them from the specific Wnt-targeting effects.^12,33^

To validate the ability of the ΔFZD_1–10_ HEK293T cell line and to accurately report responses of the individual FZD proteins, we first tested the levels of the TopFlash reporter induced in this line upon the transfection of constructs encoding individual FZDs (Figure S1A). Indeed, reexpression of individual FZDs in this line induces a 3- to 10-fold response upon addition of Wnt3a. The response reconstituted by the individual FZDs is not influenced by the EGFP tag at the N-terminus of the proteins. These results justify the applicability of this assay for the validation of FZD inhibitor compounds.

### Fz7–21 Peptide Demonstrates the Anticipated Selectivity Profile in the FZD-Selective Assay, Despite a Reduction in Potency

**Figure 2:**
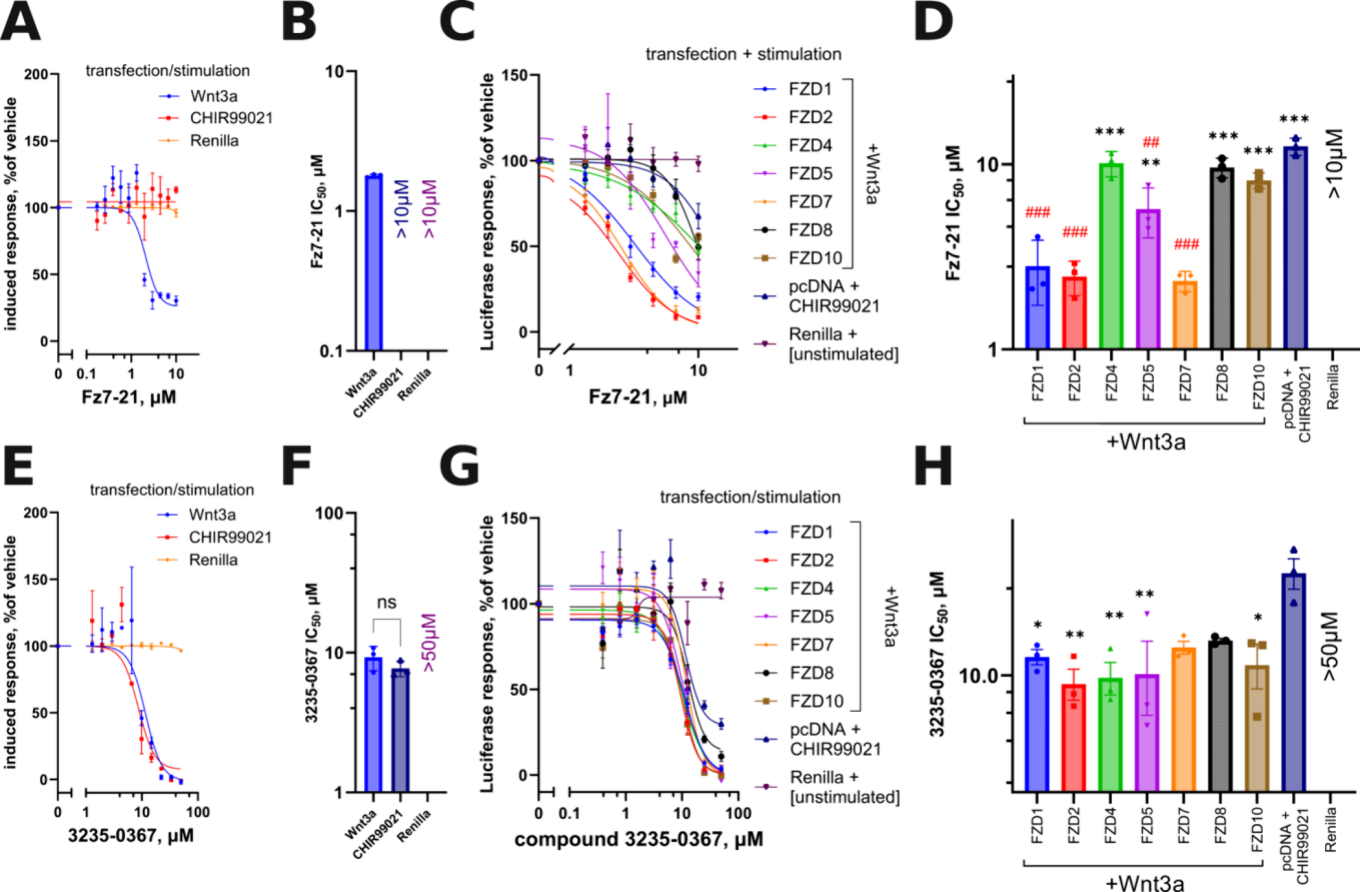
(A, C, E, G) Concentration–response curves and (B, D, F, H) IC_50_ quantifications with statistical analysis of the Fz7–21 peptide’ and the compound 3235–0367’ activities against Wnt3a- or CHIR99021-stimulated Wnt pathway activation in the wild-type reporter HEK293T line (A, B, E, F) and against individually reexpressed FZDs (_1_,_2_,_4_,_5_,_7_,_8_, and _10_) in ΔFZD_1–10_ HEK293T cells (C, D, G, H). For Fz7–21, the curves and IC_50_ values confirm the selectivity for FZD_1,2 and 7_, also reflected in the partial response in the HEK293T line. Compound 3235–0367 performs against all FZDs with a similar potency, close to that at which it exerts the nonspecific inhibition of the downstream activity of the pathway induced by CHIR99021 in the absence of FZDs, with the same pattern observed in wild-type HEK293T cells. Data in all panels are from *N* = 3 independent experiments, panels (D); (F) and (H) are analyzed by one-way ANOVA; and significance is shown as *p < 0.05, **p < 0.01, ***p < 0.001 (comparison to FZD_1_ in (D) and pcDNA+CHIR99021 in (H)) and ^##^*p* < 0.01, ^###^*p* < 0.001 (to pcDNA+CHIR99021 in (D)).

We next evaluated the selectivity profile of Fz7–21 (Figure 1), a peptide derived from a phage library and reported to target a subclass of FZD proteins^22^ including FZD_1,2, and 7_. In the original report, the peptide was functionally tested for its ability to inhibit the pathway in TopBrite HEK293 cells stimulated by Wnt3a, where it demonstrated the potency of approximately 100 nM and a nearly complete inhibition of the pathway. We first sought to reproduce these results using an in-house-derived reporter HEK293T cell line stably transfected with TopFlash (Figure 2A,B). The peptide demonstrated robust inhibition of the Wnt signaling, although its potency did not reach the reported levels, resulting in a value of around 2 μM and partial inhibition with an efficacy of around 70%, with no inhibitory activity seen when the same line is stimulated with CHIR99021, a GSK3β inhibitor that stimulates the cascade by directly stabilizing β-catenin, bypassing FZDs.

Despite the lower than reported potency, we confirm the reported selectivity profile of Fz7–21 in the ΔFZD_1–10_ HEK293T cells retransfected with FZD_1,2, and 7_, with nearly identical IC_50_s of around 2 μM against the three FZDs (Figures 2C,D and S1B). The potency of Fz7–21 against FZD_5_ is >3-fold lower and about 5-fold lower against the remaining FZDs. In the dual luciferase setting of the experiment,^34,35^ no unspecific toxicity and no general transcriptional inhibition by the Fz7–21 peptide are observed when monitoring the Renilla luciferase signal. However, the peptide at the highest technically achievable concentration (10 μM) demonstrates a surprising and statistically significant, though weak, ability to inhibit the response of the TopFlash reporter to the GSK3β inhibitor CHIR99021 in these cells transfected with the control pcDNA3.1 vector, i.e., independently of FZD. The projected IC_50_ of this effect is around 15 μM, which is comparable to the ∼10 to 11 μM potency found for FZD_4,8, and 10_. Given this effect, it is impossible to reliably evaluate the potency of Fz7–21 against these three receptors. In conclusion, apart from the unexpected significant activity against FZD_5_, the selectivity profile of Fz7–21 is in line with the published data. However, it should be noted that the potencies we obtained in HEK293T cells with native expression of FZDs, as in the ΔFZD_1–10_ HEK293T cell reexpressing individual FZD_1,2, or 7_, are about 1 order of magnitude lower than those reported in the original study.

### Small Molecule 3235-0367, Purported to be Selective for FZD_8_, Exhibits a Low Potency FZD-Independent Inhibition of the Wnt Pathway

Next, we retested the compound designated 3235-0367, which originated from the virtual screening with FZD_8_ CRD as the target.^19^ The authors employed TopFlash-transfected 3T3 cells to assess the activity of the hit compounds, asserting a potency of 7.1 μM for 3235-0367. We first attempted to replicate their results using a similar reporter cell line that natively expresses multiple FZDs, HEK293T stably transfected with TopFlash (Figures 2E,F and S1C). While 3235-0367 shows the inhibition pattern with an IC_50_ of around 8 μM, similar to the reported value,^19^ when stimulated with Wnt3a, it is obvious that this inhibition cannot be mediated by FZDs, as an overlapping inhibition curve is observed when these cells are stimulated with CHIR99021. Upon challenging 3235-0367 against individual FZDs, no significant selectivity against any FZD including FZD_8_ can be identified. The mean IC_50_ values varied between 9–11 μM, with no statistically significant differences among them (Figure 2G,H). Similarly to Fz7–21, 3235-0367 does not demonstrate any toxicity or general transcriptional inhibition up to 50 μM, as evaluated by the Renilla luciferase levels. However, as in wild-type HEK293T cells, the compound inhibits the signal induced independently of FZDs by the GSK3β inhibitor CHIR99021, with a potency of approximately 20 μM. While the downstream inhibition is statistically distinct from the identified potencies for FZD-transfected cells, these differences are marginal. When taken together with the indistinguishable activity of the compound against both modes of pathway stimulation in wild-type HEK293T cells, our findings make us conclude that 3235–0367 is not a selective FZD_8_ inhibitor, is not a pan-FZD inhibitor, but is instead an inhibitor of a certain unidentified pathway component downstream of GSK3β.

### Claimed FZD_7_-Targeting Activity of SRI35959 and the Claimed FZD_8_-Targeting Activity of Carbamazepine Are Contradicted by the Downstream Inhibition of the Pathway Induced by the Two Compounds, Which Occurs Independently of FZD Proteins

We further retested two additional proposed FZD-targeting compounds, SRI35959 and carbamazepine. SRI35959 was identified in a virtual screening against the 7TM domain of FZD_7_ and claimed to inhibit this receptor with an IC_50_ of approximately 3 μM. However, the lack of selective assays in the original study limits the reliability of this assumption, as the functional tests used wild-type HEK293 Super8XTopFlash cells stimulated by Wnt3a or LRP6 overexpression, reinforced by Western blot-based evaluation of β-catenin and Wnt target genes in 2 cancer cell lines.^18^ In a separate study, carbamazepine was identified as an interactor of CRD of FZD_8_. This was confirmed by a surface plasmon resonance (SPR) binding assay and a crystal structure. Further, in the Wnt3a-irresponsive HEK293T cell line knocked out of FZD_1,2, and 7_, where the Wnt3a response was restored by transfection with mouse FZD_8_, carbamazepine partially inhibited the Wnt3a-induced signal.^16^ However, the authors did not challenge the selectivity of the compound using downstream-induced assay activation (e.g., by CHIR99021) or upon transfection with other FZDs.

**Figure 3:**
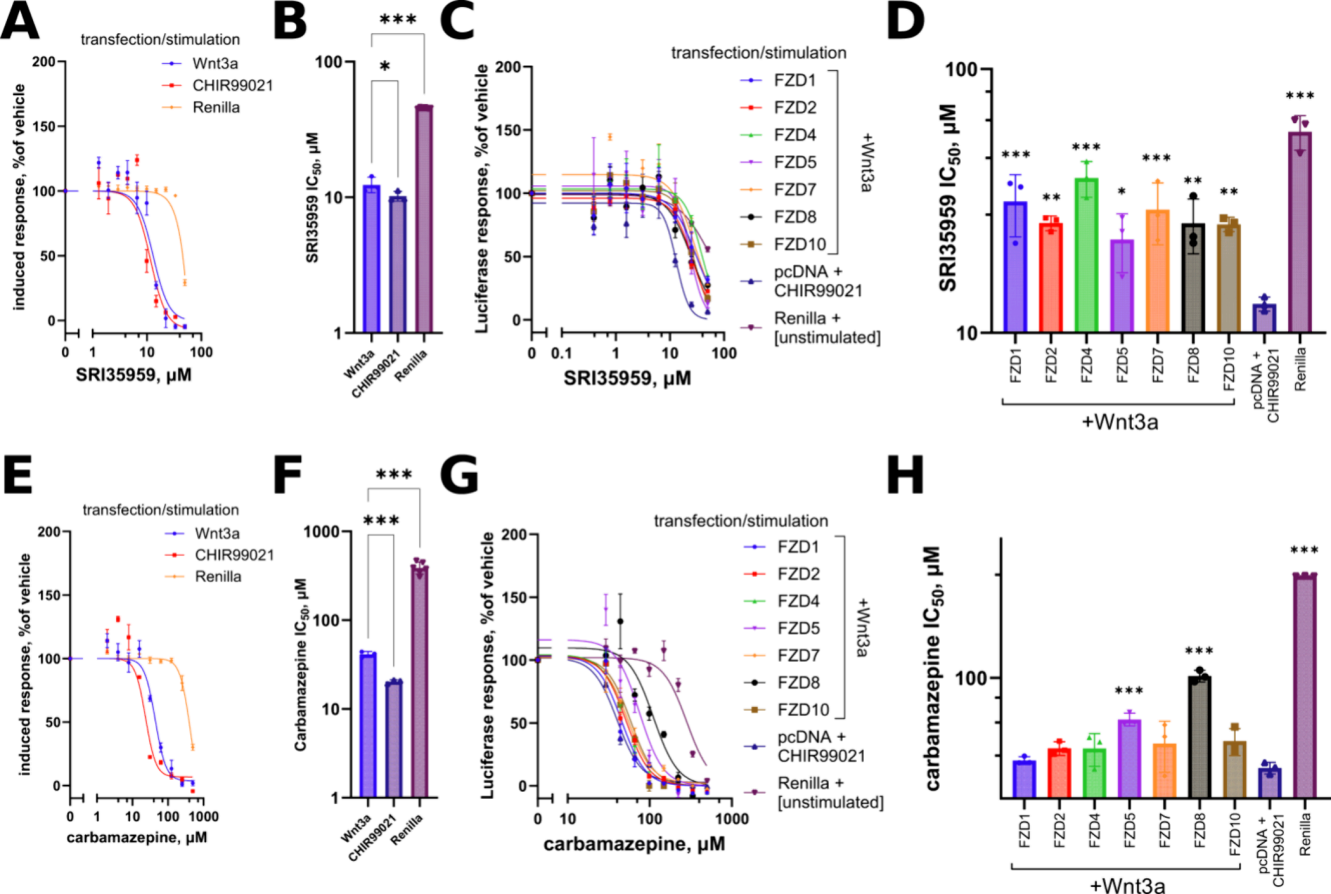
(A, C, E, G) Concentration–response curves and (B, D, F, H) IC_50_ quantifications with statistical analysis of SRI35959’ and carbamazepine’ activities against Wnt3a- or CHIR99021-stimulated response in the wild-type reporter HEK293T line and against individual FZDs reexpressed in ΔFZD_1–__10_ HEK293T cells. Both compounds show comparable potency in the FZD-dependent (by Wnt3a) and FZD-independent (by CHIR99021) activation of Wnt signaling in both cell lines. Data in all panels are from *N* = 3 independent experiments, panels (B, D, F, H) are analyzed by one-way ANOVA, and significance is shown as *p < 0.05, **p < 0.01, ***p < 0.001 (in comparison to pcDNA+CHIR99021 condition in (D) and (H)).

In our selective functional assays, we find that both SRI35959 and carbamazepine similarly inhibit the Wnt signaling induced by Wnt3a and CHIR99021 in wild-type HEK293T cells (Figures 3A,B,E,F and S1D,E). In fact, the potency of both compounds is even slightly (but significantly) better when CHIR99021 is used. To assess whether SRI35959 or carbamazepine can display any FZD-selective inhibitory activities that might be masked in wild-type HEK293T cells expressing multiple FZDs, we next analyzed the two compounds in individual FZD-reconstituted ΔFZD_1–__10_ HEK293T cells. In these cells, we find both compounds to exhibit a comparable pattern, incompatible with a specific functional inhibition of a FZD protein, FZD_7_ or _8_ as reported in the original studies, or any other, as the compounds demonstrate a robust inhibition of the pathway even in the absence of any FZD transfection (Figure 3C,D,G,H). While the IC_50_ of this CHIR99021-induced activity is 10 μM for SRI35959 and is close to the reported value, the IC_50_ of carbamazepine is observed to be approximately 50 μM or about 8–10 higher than the values reported in the TopFlash assay in the original study. The capacity of SRI35959 and carbamazepine to inhibit FZD-independent CHIR99021-induced Wnt pathway activation suggests that they too act on an as yet unidentified component(s) of the pathway located downstream of GSK3β. Interestingly, the compounds’ capacity to inhibit this downstream pathway component(s) is modulated upon reintroduction of certain FZDs: FZD_1_,_4_, _and 7_ are found to reduce the potency of SRI35959 and FZD_5_ and FZD_8_ of carbamazepine (Figure 3D,H), the effect possibly mediated by some feedback loops within the Wnt pathway (see Discussion). It should also be noted that although both compounds exhibit some toxicity as evidenced by their effect on Renilla luciferase, the potency of this effect is clearly much lower than that of the Wnt pathway inhibition (Figure 3B,D,E,F), not confounding the interpretation of the Wnt inhibitory activities.

### Inhibition of Wnt Signaling by Niclosamide Is Not Contingent on FZDs Including FZD_1_

**Figure 4:**
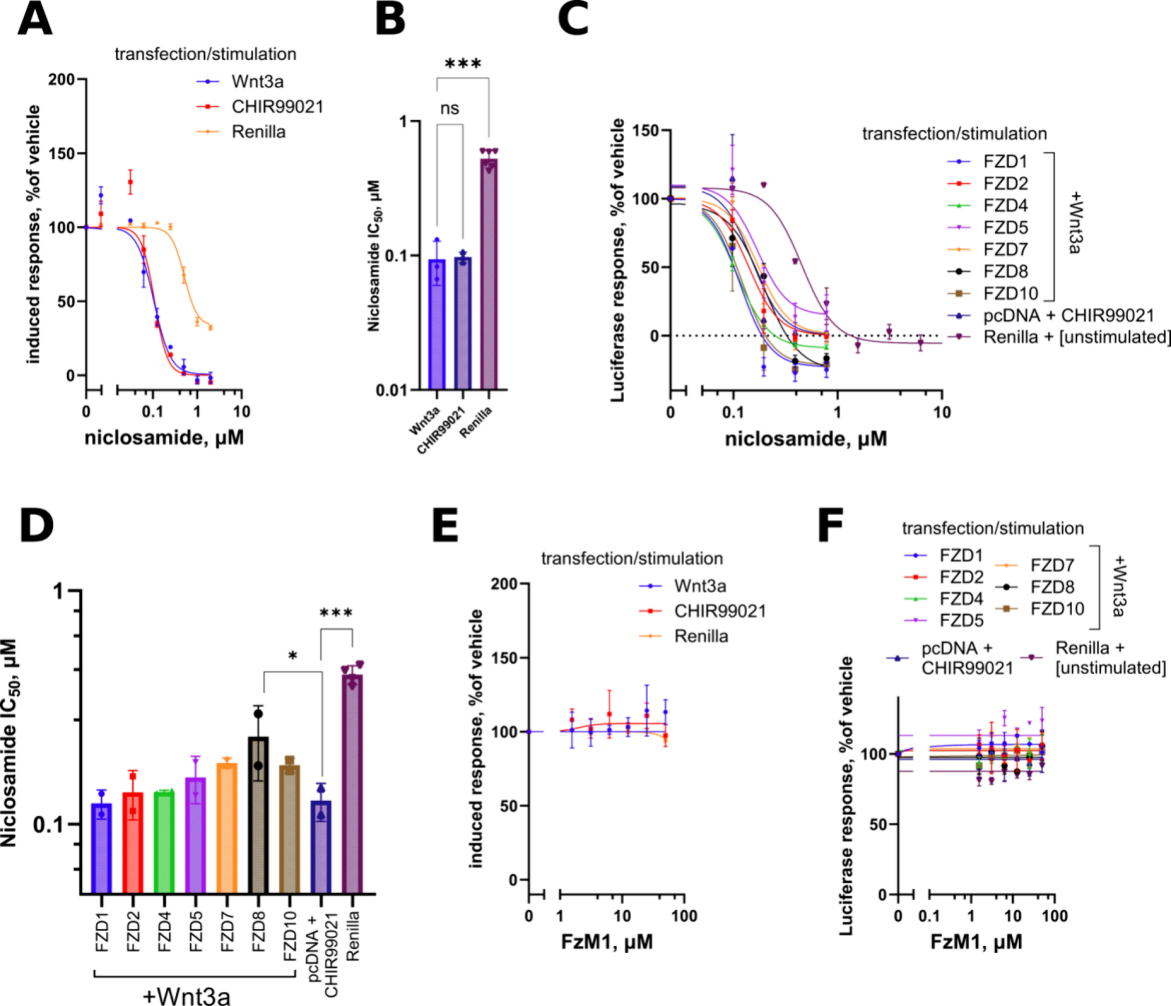
(A, C) Concentration–response curves and (B, D) IC_50_ quantifications with statistical analysis of niclosamide activity against Wnt3a- or CHIR99021-stimulated response in the wild-type HEK293T or against individually reexpressed FZDs in ΔFZD_1–__10_ HEK293T cells. Compound elicits an FZD-independent pathway inhibition. (E, F) Concentration–response curves in the same test systems of the compound FzM1 demonstrating no detectable activity against any form of Wnt signaling up to 50 μM. Data in panels (B, D) are from *N* = 2–3 independent experiments, analyzed by one-way ANOVA, and significance is shown as *p < 0.05 and ***p < 0.0001 in comparison to pcDNA+CHIR99021 condition.

Another compound which was proposed to likely act directly on FZD to block β-catenin-dependent Wnt signaling at the potency of 0.5–1 μM is niclosamide.^15^ The drug was proposed to mediate the pathway inhibition through loss of LRP6 phosphorylation^36^ and increased FZD_1_ internalization in response to Wnt3a.^15^ Although the compound was assumed to target FZD_1_ directly, no selectivity among FZD proteins was ever claimed or reported for this compound. Our verification of the compound in the wild-type HEK293T reporter line analogous to that in the original study (Figure 4A,B) or in the FZD-selective line based on ΔFZD_1–10_ HEK293T cells (Figures 4C,D and S1F) resulted in the identical (IC_50_ = 100 nM) striking inhibition of the Wnt pathway under all conditions tested, including the CHIR99021-mediated GSK3β inhibition in the cells deprived of any FZD protein. We thus must conclude that the target of niclosamide is a Wnt pathway component lying downstream of the β-catenin destruction complex. Interestingly, the compound also possesses a quite potent unspecific activity against these cells shown as inhibition of Renilla signal, with a potency of around 0.5 μM.

### FzM1, Reported as a Negative Allosteric Modulator Selective for FZD_4_, Does Not Affect FZD_4_-Dependent or Any Other Wnt Pathway Activity Induced by Wnt3a

A small molecule compound called FzM1 was studied as a negative allosteric modulator (NAM) of FZD_4_.^21^ In the original study, no FZD selectivity was assessed and the compound itself was evaluated to target basal activity of FZD_4_ resulting from its overexpression, as well as coexpression with LRP5 and stimulation by Norrin in wild-type HEK293 cells. It should be emphasized that in their reporter HEK293 cell line, the compound was only able to inhibit the Norrin-induced activity of FZD4, with no effect on the pathway stimulated by simultaneous overexpression of FZD4 and LRP5, which can be considered as a surrogate for Wnt ligand stimulation. We investigated whether the reported NAM effect could manifest upon Wnt3a activation, as this is of considerable interest for evaluation of the perspectives of this compound. However, no statistically discernible effect of the compound was identified either in wild-type HEK293T cells or against any of the FZDs in ΔFZD_1–__10_ HEK293T, including FZD_4_ (Figures 4E,F and S1G).

### Derivative #28 of F7H (F7H-28), a Compound Proposed to Act as a FZD_7_ Antagonist, Exhibits Potent Inhibitory Activity against Firefly Luciferase without Affecting Wnt Signaling

**Figure 5:**
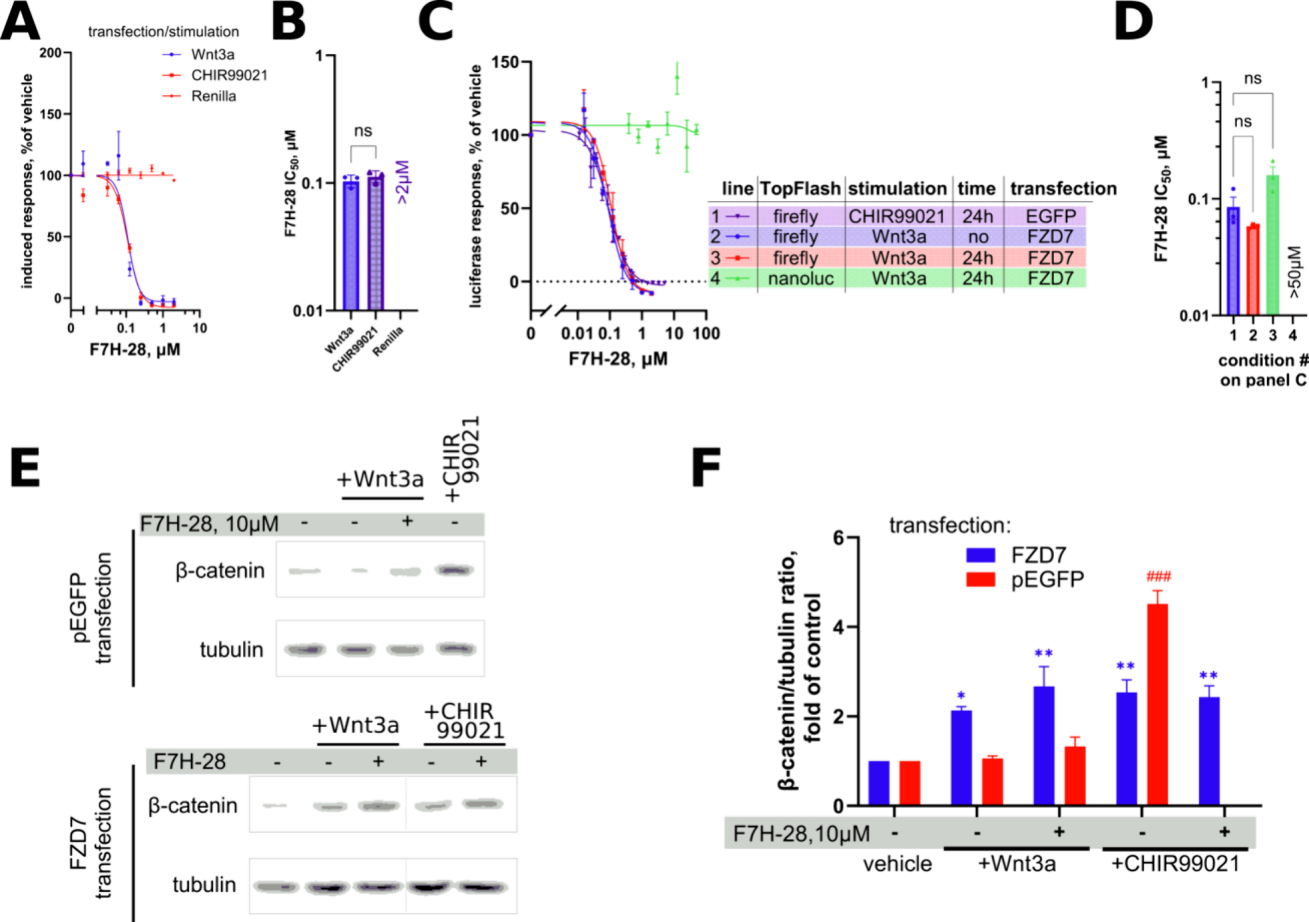
(A, C) Concentration–response curves of compound F7H-28 in different assay conditions, including TopFlash reporter wild-type HEK293T and TopFlash with NanoLuc instead of Firefly as the Wnt reporter gene in ΔFZD_1–__10_ HEK293T cells. (B, C) Quantification of IC_50_ values from these curves. Data show that F7H-28 is capable of signal suppression when induced by the GSK3β inhibitor or Wnt3a, even when added directly before cell lysis, but does not suppress the response of the FZD_7_-induced NanoLuc reporter, proving that the compound inhibits the firefly luciferase enzymatic activity. (E) FZD_7_ transfection allows one to observe an increase in cytoplasmic β-catenin levels in ΔFZD_1–__10_ HEK293T cells in response to Wnt3a, but treatment of the cells with F7H-28 does not reduce this signal. Western blot signal quantification and statistical evaluation are shown in (F). Data in panels (B, D, F) are from *N* = 3 independent experiments and were analyzed by one-way ANOVA, and the significance is shown as *p < 0.05, ***p < 0.01, ^###^*p* < 0.001.

Virtual screening using the modeled structure of the 7TM domain of FZD_7_ as a target identified one compound, F7H, as a binder to this domain and an inhibitor of TopFlash stimulated in HEK293T cells by cotransfection with a Wnt3a-expressing construct.^20^ A most potent derivative reported in this study (no. 28, referred to as F7H-28 in the present work) was resynthesized with a reported IC_50_ as low as 40 nM. Upon retesting the compound in wild-type HEK293T cells as well as in ΔFZD_1–__10_ HEK293T cells without any FZD reexpression stimulated with CHIR99021, we observe a strong concentration-dependent inhibition of the TopFlash signal. The same potency of the compound is observed when Wnt3a is used to stimulate the cells upon FZD_7_ reexpression (Figure 5A–D). Curiously, treatment with F7H-28 results in a drop in the luciferase readings below the values observed in unstimulated cells (as evidenced by the drop of the normalized response curves below the zero level, as illustrated in Figure 5A,C; also see Figure S1H). This unusual behavior prompted us to investigate whether the compound had a direct effect on firefly luciferase. As demonstrated by the curve obtained upon addition of the compound F7H-28 to the cells that had been prestimulated immediately prior to lysis and measurement (Figures 5C and S1H), one can exclude the possibility of reduction in levels of the accumulated luciferase due to the shutdown of the Wnt response. Instead, a direct effect of the compound on firefly luciferase is suspected. Further support for this hypothesis comes from our experiment where we used a Wnt reporter system employing the NanoLuc enzyme in place of the firefly luciferase. This reporter is fully capable of monitoring the response to Wnt3a stimulation when cotransfected with FZD_7_ but does not report significant changes when F7H-28 is added (Figures 5C and S1H). Furthermore, we could further substantiate the lack of a discernible effect of F7H-28 on FZD_7_ signaling through the examination of Wnt3a/FZD_7_-mediated cytoplasmic β-catenin accumulation by Western blotting (Figure 5E,F).

## Discussion and Conclusions

The targeting of FZDs has long been regarded as a privileged mode of drug development, not only for compounds targeting Wnt signaling but also for Wnt-activating therapies in the field of stem cell and regenerative medicine.^7,8^ This is primarily due to the potential to achieve unparalleled selectivity of such agents, as 10 FZD members are differentially expressed across various tissues and cell types. The present study sought to address a significant limitation of previous research on selective FZD inhibitors, namely, the lack of rigorous functional validation of their selectivity. This has led to an over-reliance on physical binding patterns or in silico data to infer the selectivity profile of a given molecule. By establishing a selective functional assay using ΔFZD_1–__10_ HEK293T cells,^26^ we were able to directly assess the ability of reported compounds to inhibit the canonical Wnt signaling pathway mediated by individual FZD proteins, including those compounds claimed to be selective FZD inhibitors.

The generation of the ΔFZD_1–__10_ HEK293T cell line marks a major advancement in the development of selective Wnt-targeting agents. The elimination of all 10 FZD proteins in these cells provides a genetically pure background, allowing for the study of the functional activity of individual FZD proteins upon their reexpression. This finally allows the functional evaluation of individual FZDs in a manner comparable to other GPCRs, which typically exhibited more exclusive expression patterns, and for which the identification of cell lines without native expression did not represent a significant challenge.^27^ In contrast, FZDs are present in virtually every cell type, as shown by the results of systematic sequencing campaigns in the model cell lines.^23−25,37^ Consequently, even overexpression of a FZD on a background of native expression patterns provides a limited research opportunity.^29^ Only the genetic knockout of all ten FZDs, combined with reexpression of a desired individual receptor, now allows the clean assessment of the selectivity profile of a given inhibitory compound. Further, the use of the ΔFZD_1–__10_ cell line in conjunction with the downstream pathway activation with CHIR99021 (a GSK3β inhibitor) provides an unparalleled control over the compounds’ mode of action. This toolkit permitted us to functionally validate the claimed FZD inhibitors in the β-catenin-dependent Wnt signaling—the “Holy Grail” of many modern pharmaceutical programs. We note that any noncanonical, β-catenin-independent branches of the Wnt pathway remained outside of our current study, as they were in the original reports describing the FZD inhibitors.

The results of our study demonstrate that the majority of previously reported FZD inhibitors are ineffective in functional inhibition of any FZD in the canonical Wnt signaling pathway. Furthermore, if these compounds can inhibit the Wnt pathway at all, they do so via targeting unknown component(s) of the pathway acting downstream of GSK3β. Such FZD-independent activities emerge for carbamazepine and 3235-0367 (claimed to be a FZD_8_-selective compounds^16,19^), compound SRI35959 (claimed to target FZD_7_^18^), and niclosamide (previously believed to act on FZD_1_^15^). Of these compounds, 3235-0367 could be suspected, in addition to its ability to inhibit some downstream pathway component, to possess pan-FZD inhibitor properties, as it demonstrated a small yet statistically significant difference in its activity against Wnt3a-stimulated reexpressed FZDs and the GSK3β inhibitor-derived signal. However, these results are in contrast to those obtained for the Wnt3a- and CHIR99021-induced responses in wild-type HEK293T cells, where both readouts were identically inhibited by 3235-0367. These variations are likely due to the unique characteristics of the knockout cell line, which may have arisen during clonal selection or due to chronic absence of FZDs, such as reduction/relocation of the compound-responsive downstream pathway component, reconstituted upon FZD overexpression.

Similarly, it is noteworthy that some of the aforementioned compounds inhibit CHIR99021-induced pathway activation in FZD-deficient cells more potently than they do upon Wnt3a stimulation in some FZD-reconstituted cells. Thus, FZD_1_,_4_, and _7_ reexpression apparently reduces the potency of SRI35959 and FZD_5_ and FZD_8_ of carbamazepine. We can speculate that these effects may reflect certain feedback regulations within the Wnt signaling such that some FZDs are capable of eliciting regulatory effects on the downstream components–targets of carbamazepine and SRI35959, which may be analogous to their differential capacity to engage different G proteins.^38−40^

Among the compounds we studied, FzM1 was previously not evaluated for functional activity in assays driven by Wnt3a.^21^ Instead, it was tested only for a basal, Norrin-mediated, or LRP overexpression-induced activity. In our assays, overexpression of individual FZD proteins does not result in significant ligand-independent pathway activation. However, it is evident that FzM1 is incapable of influencing FZD_4_-mediated or, indeed, any other FZD-mediated signaling cascades initiated by Wnt3a stimulation. An even more unexpected finding from our experiments was that the most potent F7H derivative, F7H-28,^20^ is not a selective inhibitor of FZD_7_ but rather a compound that selectively and potently inhibits firefly luciferase, with no discernible activity against FZD_7_ in either a reporter-based or an orthogonal Western blotting-based analysis.

Our work confirms a single agent, the peptide designated Fz7–21, as a reliable compound with functional selectivity against FZD_1_,_2_, and _7_.^22^ This finding is consistent with the structural and in vitro binding profiling of this compound in the original study; however, we report significantly (3–5-fold) lower potency of this peptide. This could be the result of different synthetic routes and purity but is most likely related to the cellular context, particularly in terms of the proteolytic activity and peptide scavenging. As in the original studies describing the Wnt-targeting compounds, we utilized Wnt3a to stimulate FZD-dependent signaling. Regarding Fz7–21 as the only FZD inhibitor we could confirm, it might be interesting to investigate in the future whether its potency and receptor selectivity might change upon pathway activation with another Wnt family member. These experiments might enlighten the drug development perspectives of Fz7–21.

The results of our study highlight the necessity of careful functional validation in the process of drug discovery. While physical binding patterns and in silico data can provide valuable insights, they do not necessarily correlate with the actual inhibitory activity. In fact, our functional findings do not even contradict the results of the physical binding assays. We also cannot fully exclude that some of these compounds, at concentrations above those needed to inhibit their downstream Wnt pathway targets, are also FZD inhibitors. In this unlikely scenario, their FZD inhibition does not make any functional importance, as they are first of all now proven to be downstream pathway inhibitors. However, if true binders, some of these compounds might be developed further into true *functional* inhibitors of the respective receptors^41^—if and when the proper functional assays are an integral part of the respective drug development campaign.

In conclusion, our study illustrates the critical importance of utilizing stringent and selective functional assays in an appropriate manner to assess the efficacy of screening-derived compounds as prospective FZD inhibitors. By addressing the shortcomings of the previous research, our findings contribute to a more precise understanding of the challenges and opportunities in the development of selective FZD inhibitors for therapeutic applications. We postulate that our work will also instruct many future Wnt-targeting drug discovery campaigns.

## Experimental Section

### Generation of the Stably Transfected by TopFlash Reporter ΔFZD_1–10_ HEK293T Line and FZD Reexpression by the BacMam System

The ΔFZD_1–10_ HEK293T were a kind gift from Prof. Vanhollebeke and were cultured as described.^26^ The cells were stably transduced by lentivirus encoding a firefly luciferase-based TopFlash cassette recloned from the TopFlash M50 vector (#12456)^42^ into the pLenti backbone (Addgene #39481).^43^ At 2 days postinfection, cells were selected for puromycin resistance and established into a line. For FZD reintroduction, the human FZD_1,5,7 and 10_ and mouse Fzd_2,4, and 8_ were cloned into the pEG BacMam vector (Addgene #160451)^44^ with N-terminal EGFP tags and mPrP leader peptide, and the corresponding baculoviruses were harvested according to standard protocol in DH10Bac *E. coli*. Baculovirus was produced in SF9 cells cultured in a TNM-FH medium. Baculovirus supernatants were used to infect cultured TopFlash reporter ΔFZD_1–10_ HEK293T in the presence of 10 mM sodium butyrate. For toxicity evaluation, reporter ΔFZD_1–10_ HEK293T were transfected with the Renilla luciferase under the CMV promoter encoding plasmid (pRL-CMV) using 12 μg/mL of DNA and 40 μL/mL of XtremeGENE 9 reagent overall following the manufacturer’s instructions,^35^ and drops in the Renilla levels were further confirmed by microscopically assessed cytotoxicity. An identical transfection protocol was used to transfect untagged and EGFP-tagged constructs encoding the listed FZDs. Untagged versions were cloned in pcDNA FZD_1,5,7, and 10_,^45^ and in pRK5 for mouse Fzd_2_ (Addgene #42254), _4_ (Addgene #42256), and _8_ (Addgene #42260);^46^ GFP-tagged all cloned in pEGFP plasmid modified with mPrP leader sequence. To generate NanoLuc substituted TopFlash, the firefly insert was excised from the TopFlash M50 vector, and the NanoLuc luciferase coding sequence was inserted using the Gibson assembly method.

### TopFlash Assay in Reporter ΔFZD_1–10_ HEK293T Line

Wnt3a- or CHIR99021-stimulated luciferase activity in TopFlash reporter-stably transfected ΔFZD_1–__10_ HEK293T cells with reintroduced FZDs was assayed in a manner similar to BT-20 or HCC1395 TNBC cell lines.^12,14^ In a white opaque 384-well plate, reporter cells infected or transfected as described in the previous section were seeded at a density of 10,000 cells per well with a final volume of 20 μL of DMEM:F12 medium supplemented with 10% FCS and allowed to attach overnight at 37 °C in 5% CO_2_. The next day, 10 μL of fresh medium containing the compound of interest at the appropriate dilution was added to each well. After preincubation of the compounds for 1 h, another 10 μL of medium supplemented with 500 ng/mL Wnt3a (purified as described^45^) or 1 μM CHIR99021 was added. Compound dilutions were made by serial dilution in DMSO, and the concentration of DMSO was maintained at 0.5% throughout the test points, including the controls. After an overnight incubation period, the supernatant of each well was removed and the luciferase activity was measured as follows: the culture medium was completely removed with a washer-dispenser, and then, the luciferase activity of the firefly, Renilla, or NanoLuc luciferases was sequentially detected in each well of a 384-well plate by injecting the corresponding measurement solutions^12^ and immediately reading the results (with an integration time of 400 ms) in the Infinite M Plex M200 multifunctional plate reader with injection module.

### TopFlash Assay in a Reporter Wild-Type HEK293T Line

The HEK293T cells were stably transfected with the TopFlash M50 vector mixed with the puromycin resistance encoding plasmid pPuro. Three puromycin-resistant single colonies, selected for the strongest response to added Wnt3a, were mixed and established as a reporter cell line. The overall pathway activity of these lines was evaluated identically to that of the ΔFZD_1–10_ HEK293T line, except that instead of FZD transduction by BacMam vectors, they were transfected with the pRL-CMV encoding plasmid.

### Western Blot-Based Analysis of FZD7-Dependent Accumulation of β-Catenin

In 24-well plates, a ΔFZD_1–__10_ HEK293T cell line was reinfected with a baculovirus expressing FZD7 or a control baculovirus at a density of 100,000 cells/well. The new medium containing the 10 μM F7H-28 derivative and the indicated Wnt activators (500 ng/mL Wnt3a or 1 μM CHIR99021) was added and incubated until the next day. The medium was then removed, and 500 μL of 1× PBS was used twice for washing. After adding 30 μL of ice-cold 1× TBS supplemented with 4 mM EDTA and 1× protease inhibitor cocktail, the cells were lysed by scraping and passed through an insulin 30G needle 10 times. The samples were centrifuged at 18,000*g* and 4 °C for 10 min to remove debris. Western blot was performed on 15 μL of each supernatant and developed using antibodies against α-tubulin and β-catenin.

### Compound Sources and Synthesis

The available compounds (niclosamide, carbamazepine, SRI35959, and 3235-0367) were purchased from the respective suppliers, SigmaAldrich or Molport. FzM1 was kindly provided by Prof. Mariano Stornaiuolo. The Fz7–21 peptide (LPSDDLEFWCHVMY) was synthesized by GenScript using standard quality synthesis with purity >85%. Compound F7H-28 was synthesized in-house, as described in Supporting Information (Scheme 1 and Method 1). Compound purity was >95%, as determined by HPLC.

## Supporting Information Available

Synthesis scheme and detailed synthetic procedures and characterization of compound F7H-28 and data on the responses of the untagged and N-terminally EGFP-tagged FZD constructs and non-normalized TopFlash counts referring to the graphs (PDF)
