# Supplementary material for: Challenging Reported Frizzled-Targeting Compounds in Selective Assays Reveals Lack of Functional Inhibition and Claimed Profiles
Source: ACS Pharmacol Transl Sci. 2024 Dec 2;7(12):4144–54. doi: 10.1021/acsptsci.4c00570 (PMC11650735; doi:10.1021/acsptsci.4c00570)
Supplement: Supplementary file 1 — pt4c00570_si_001.pdf [file pt4c00570_si_001.pdf]

Supporting information for:

Challenging reported Frizzled-targeting compounds in selective assays reveals lack of functional inhibition and claimed profiles.

Alexey Koval*, Cédric Boudou, Vladimir L. Katanaev*

Department of Cell Physiology and Metabolism, Translational Research Centre in Oncohaematology, Faculty of Medicine, University of Geneva,1206 Geneva, Switzerland

*Corresponding Authors: Alexey.Koval@unige.ch, Vladimir.Katanaev@unige.ch

Contents:

F7H-28 synthesis scheme – Page S-2

Detailed synthetic procedures and characterization of compound F7H-28 – Page S-3

Figure S1 – Page S-11

Scheme 1 | Synthesis of F7H analog compound F7H-28[a]

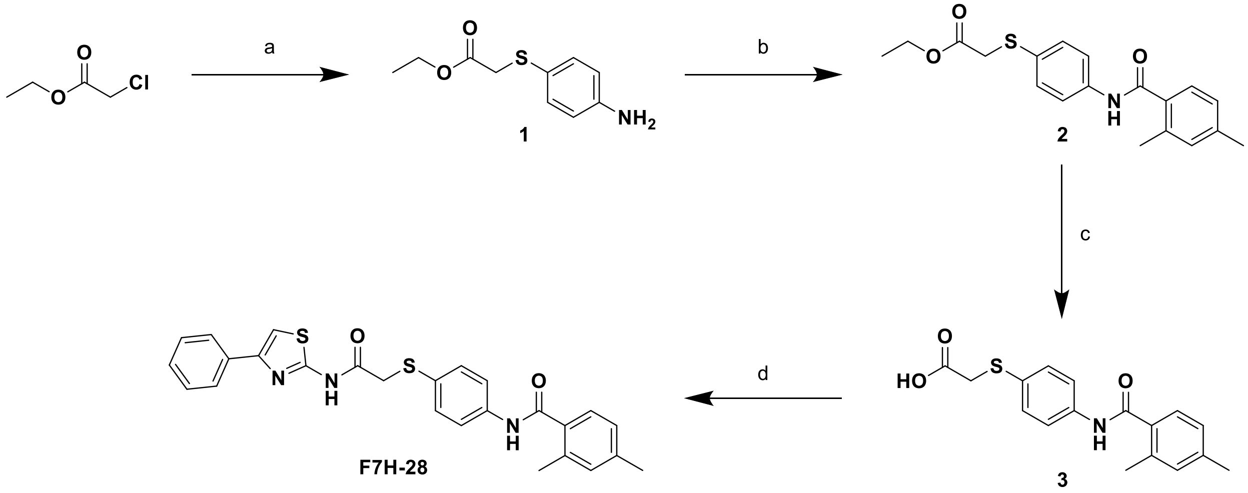

[a]Reagents and conditions: (a) 4-aminobenzenethiol, K_2CO_3, DMF, r.t., 4 h, yield 31%; (b) 2,4-dimethylbenzoic acid, HATU, DIPEA, DMF, 60°C, 12 h, yield 34%; (c) 10% KOH, MeOH, r.t., 2 h, yield 92%; (d) 4-phenylthiazol-2-amine, HATU, DIPEA, DMF, 60°C, 12 h, yield 13%.

Synthesis

1 Compound synthetic procedures and characterization of compound F7H-28

Reactions were carried out under a positive argon atmosphere unless otherwise stated. Anhydrous solvents were kept over 4Å molecular sieves. Solvents of analytical grade were used for purifications and reaction treatments. Chromatographic separations were performed on an automated Büchi Laboratory® C-815 Flash apparatus equipped with UV detector and using a pre-packed silica gel column (FlashPure; 35-45 µm). A Bruker® Avance III HD 600 MHz NMR spectrometer equipped with a QCI 5mm Cryoprobe and a SampleJet automated sample changer (Bruker® BioSpin, Rheinstetten, Germany) was used to perform 1H and 13C NMR experiments. All chemical shifts are expressed in parts per million (ppm) relative to the peak of chloroform (Chloroform-*d*; 7.26 ppm for 1H; 77.16 ppm for 13C), or deuterium methanol (MeOD; 3.31 ppm for 1H; 49.00 ppm for 13C), or dimethyl sulfoxide (DMSO; 2.50 ppm for 1H; 39.52 ppm for 13C). The protons peaks are described according to NMR conventions: “s” for singlet, “br” for broad singlet, “d” for doublet, “t” for triplet, and “q” for quadruplet. The carbon peaks are described using DEPTQ experiment which results in positive pics for quaternary “C” and “CH_2” carbons and negative pics for “CH” and “CH_3”. Unless stated otherwise carbon pics are assigned for one carbon. Chemical shifts are in ppm and constant coupling are expressed in Hz. The device used to record mass spectra was an Advion Expression® CMS operating in electrospray positive and negative mode (ESI) simultaneously.

1.1 General procedure for the synthesis of compound 4, F7H-28

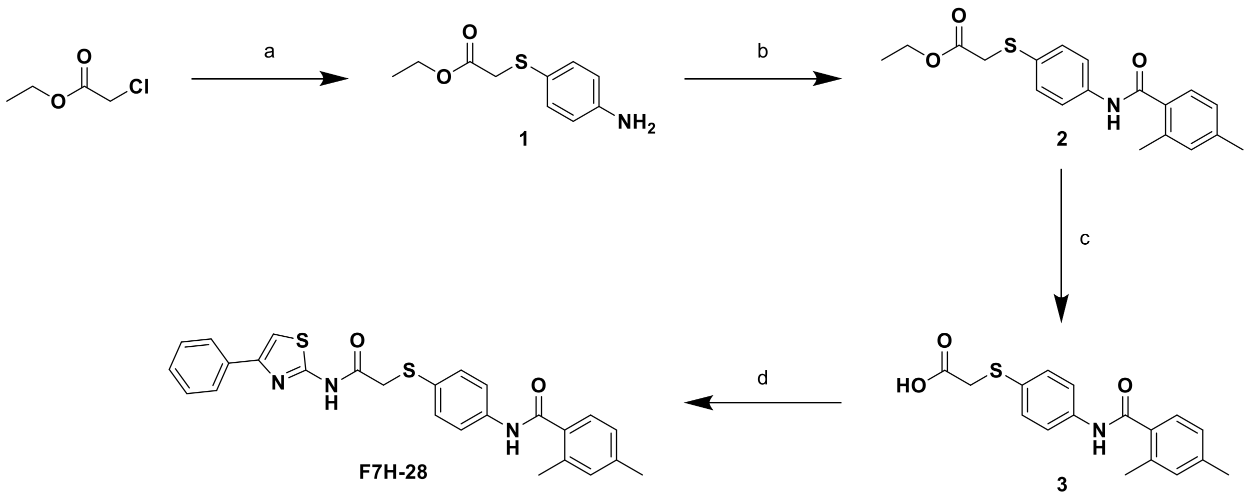

Ethyl 2-((4-aminophenyl)thio)acetate, 1

4-Aminobenzenethiol (1000 mg, 7.99 mmol, 1 eq) in dry DMF (10 mL) was added to a solution of ethyl 2-chloroacetate (870 µL, 9.59 mmol, 1.2 eq) and K_2CO_3 (1.1 g, 7.99 mmol, 1 eq) in dry DMF (16.6 mL). The resulting mixture was stirred at room temperature for 4 h. A saturated aqueous solution of $NaHCO_3$ was added to the reaction mixture and was extracted with AcOEt (3 x 40 mL). The combined organic layers were washed with a saturated aqueous solution of NaCl (3 x 20 mL) then dried over $MgSO_4$ and concentrated under reduced pressure. The crude mixture was purified by column

chromatography on silica gel using as eluent a gradient of CyHex/AcOEt from 8:2 to 6:4 to afford the compound 1 as a yellow oil (211 mg, 31%). m/z for $C_{10}H_{13}NO_2S$= 211.07 (calculated), 212.1 (found, $[M+H]^+$) and 234.1 (found, $[M+Na]^+$). ^1H NMR (600 MHz, DMSO-d6) δ 7.14 – 7.07 (m, 2H), 6.55 – 6.47 (m, 2H), 5.32 (s, 2H), 4.02 (q, J = 7.1 Hz, 2H), 3.47 (s, 2H), 1.12 (t, J = 7.1 Hz, 3H).

Ethyl 2-((4-(2,4-dimethylbenzamido)phenyl)thio)acetate, 2

To a solution of ethyl 2-((4-aminophenyl)thio)acetate, 1 (267.6 mg, 1.27 mmol, 1 eq) and 2,4-dimethylbenzoic acid (209.2 mg, 1.39 mmol, 1.1 eq) in dry DMF (6.3 mL) was added HATU (963.1 mg, 2.53 mmol, 2 eq) and DIPEA (440 µL, 2.53 mmol, 2 eq). The resulting mixture was stirred at 60°C for 12 h. A saturated aqueous solution of $NaHCO_3$ was added to the reaction mixture and was extracted with AcOEt (3 x 40 mL). The organic layer was washed with a saturated aqueous solution of NaCl (3 x 20 mL) then dried over $MgSO_4$ and concentrated under reduced pressure. The crude mixture was purified by column chromatography on silica gel using as eluent a gradient of CyHex/AcOEt from 95:5 to 80:20 to afford the compound 2 as a colorless oil (151.4 mg, 34%). m/z for $C_{19}H_{21}NOS$= 343.12 (calculated), 344.2 (found, $[M+H]^+$) and 366.2 (found, $[M+Na]^+$). ^1H NMR (600 MHz, DMSO-d6) δ 10.27 (s, 1H), 7.71 (d, J = 8.3 Hz, 2H), 7.40 – 7.33 (m, 3H), 7.15 – 7.07 (m, 2H), 4.08 (q, J = 7.1 Hz, 2H), 3.79 (s, 2H), 2.35 (s, 3H), 2.33 (s, 3H), 1.15 (t, J = 7.1 Hz, 3H).

2-((4-(2,4-dimethylbenzamido)phenyl)thio)acetic acid, 3

To a solution of ethyl 2-((4-(2,4-dimethylbenzamido)phenyl)thio)acetate (151.4 mg, 0.44 mmol, 1 eq) in mixture of water (0.2 mL) and MeOH (2.2 mL) was added slowly a 10% aqueous solution of KOH (2.5 mL, 4.41 mmol, 10 eq). The resulting mixture was stirred at room temperature for 2 h. A 1N aqueous solution of HCl was added to the reaction mixture until pH < 5 and was extracted with AcOEt (3 x 30 mL). The organic layer was washed with a saturated aqueous solution of NaCl (10 mL) then dried over $MgSO_4$ and concentrated under reduced pressure to afford compound 3 as a white solid (128.6 mg, 92%). m/z for $C_{17}H_{17}NO_3S$= 315.09 (calculated), 316.5 (found, $[M+H]^+$) and 338.5 (found, $[M+Na]^+$). ^1H NMR (600 MHz, DMSO-d6) δ 12.68 (s, 1H), 10.24 (s, 1H), 7.69 (d, J = 8.4 Hz, 2H), 7.37 – 7.33 (m, 3H), 7.13 – 7.07 (m, 2H), 3.71 (s, 2H), 2.34 (s, 3H), 2.32 (s, 3H).

2,4-dimethyl-N-(4-((2-oxo-2-((4-phenylthiazol-2-yl)amino)ethyl)thio)phenyl)benzamide, F7H-28

To a solution of 2-((4-(2,4-dimethylbenzamido)phenyl)thio)acetic acid (128.6 mg, 0.61 mmol, 1 eq) and 4-phenylthiazol-2-amine (118 mg, 0.67 mmol, 1.1 eq) in dry DMF (3 mL) was added reagent HATU (462.8 mg, 1.22 mmol, 2 eq) and DIPEA (210 µL, 1.22 mmol, 2 eq). The resulting mixture was stirred at 60°C for 12 h. As the conversion was low, HATU (231 mg, 0.61 mmol, 1 eq) and DIPEA (100 µL, 0.61 mmol, 1 eq) were added and the resulting mixture was stirred at 60°C for additional 24 h. A saturated aqueous solution of $NaHCO_3$ was added to the reaction mixture and was extracted with AcOEt (3 x 40 mL). The organic layer was washed with a saturated aqueous solution of NaCl (3 x 20 mL) then dried over $MgSO_4$ and concentrated under reduced pressure. The crude mixture was purified by column chromatography on silica gel first using as eluent a gradient of using as eluent a gradient of CyHex/EtOAc from 80:20 to 60:40 then using as eluent a gradient of CyHex/DCM from 100:0 to 90:10 to afford F7H-28 as a yellow solid (38.8 mg, 13%). m/z for $C_{26}H_{23}N_3O_2S_2$= 473.12 (calculated), 474.3 (found, $[M+H]^+$) and 496.2 (found, $[M+Na]^+$). ^1H NMR (600 MHz, DMSO-d6) δ 12.46 (s, 1H), 10.27 (s, 1H), 7.93 – 7.86 (m, 2H), 7.71 (d, J = 8.3 Hz, 2H), 7.65 (s, 1H), 7.47 – 7.39 (m, 4H), 7.38 – 7.30 (m, 2H), 7.14 – 7.07 (m, 2H), 3.92 (s, 2H), 2.34 (s, 3H), 2.32 (s, 3H). ^{13}C NMR (151 MHz, DMSO-

d6) δ 167.87, 167.59, 157.76, 148.92, 139.32, 138.46, 135.41, 134.21, 134.14, 131.18, 130.53, 128.75, 128.50, 127.84, 127.39, 126.07, 125.67, 120.16, 108.29, 37.29, 20.81, 19.35.

2 NMR spectrums

Ethyl 2-((4-aminophenyl)thio)acetate, 1

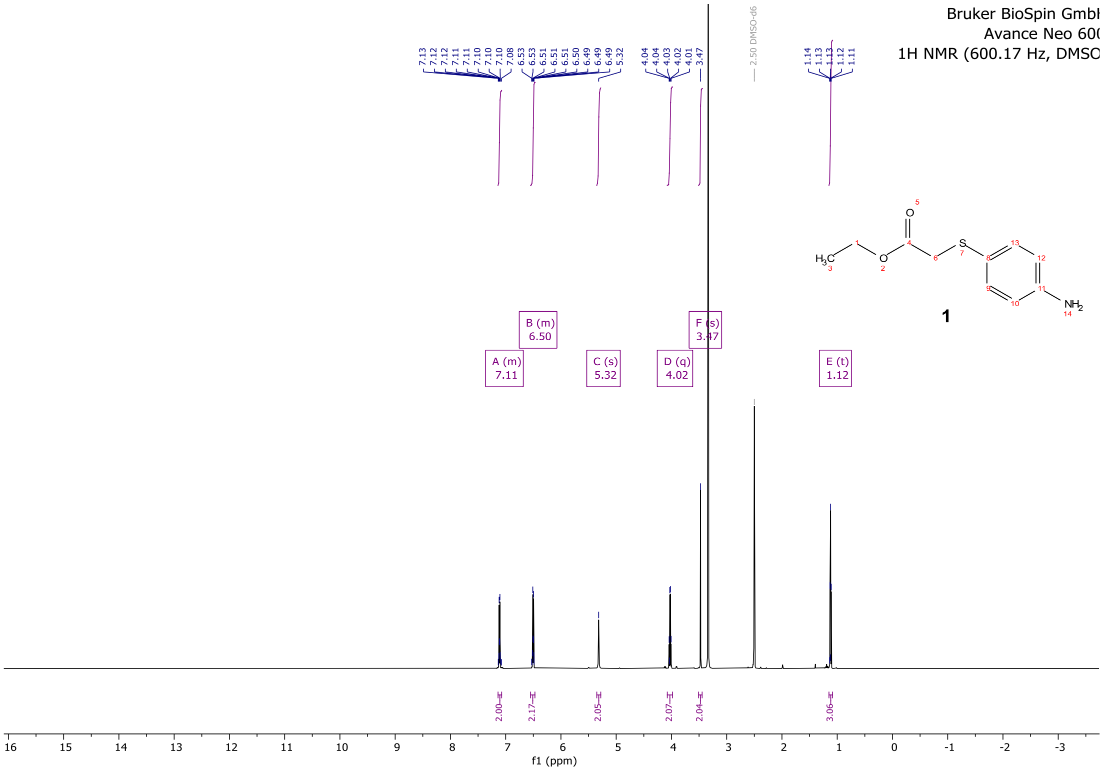

Ethyl 2-((4-(2,4-dimethylbenzamido)phenyl)thio)acetate, 2

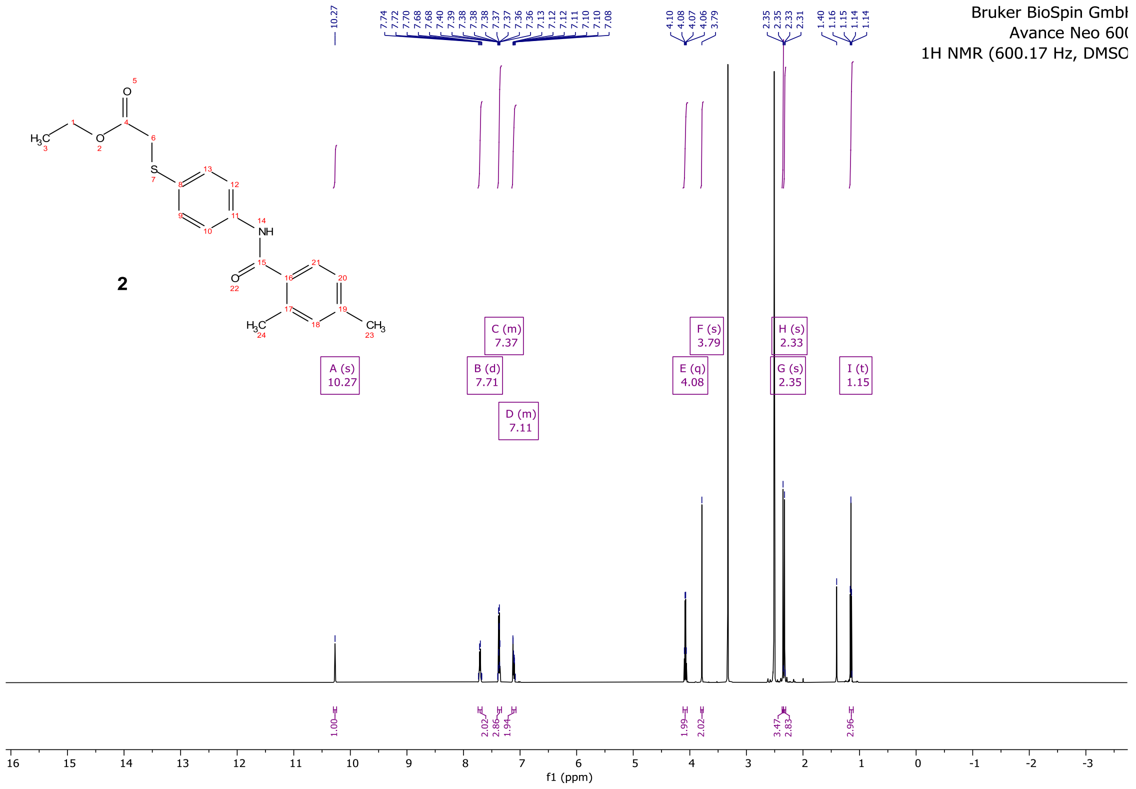

2-((4-(2,4-dimethylbenzamido)phenyl)thio)acetic acid, 3

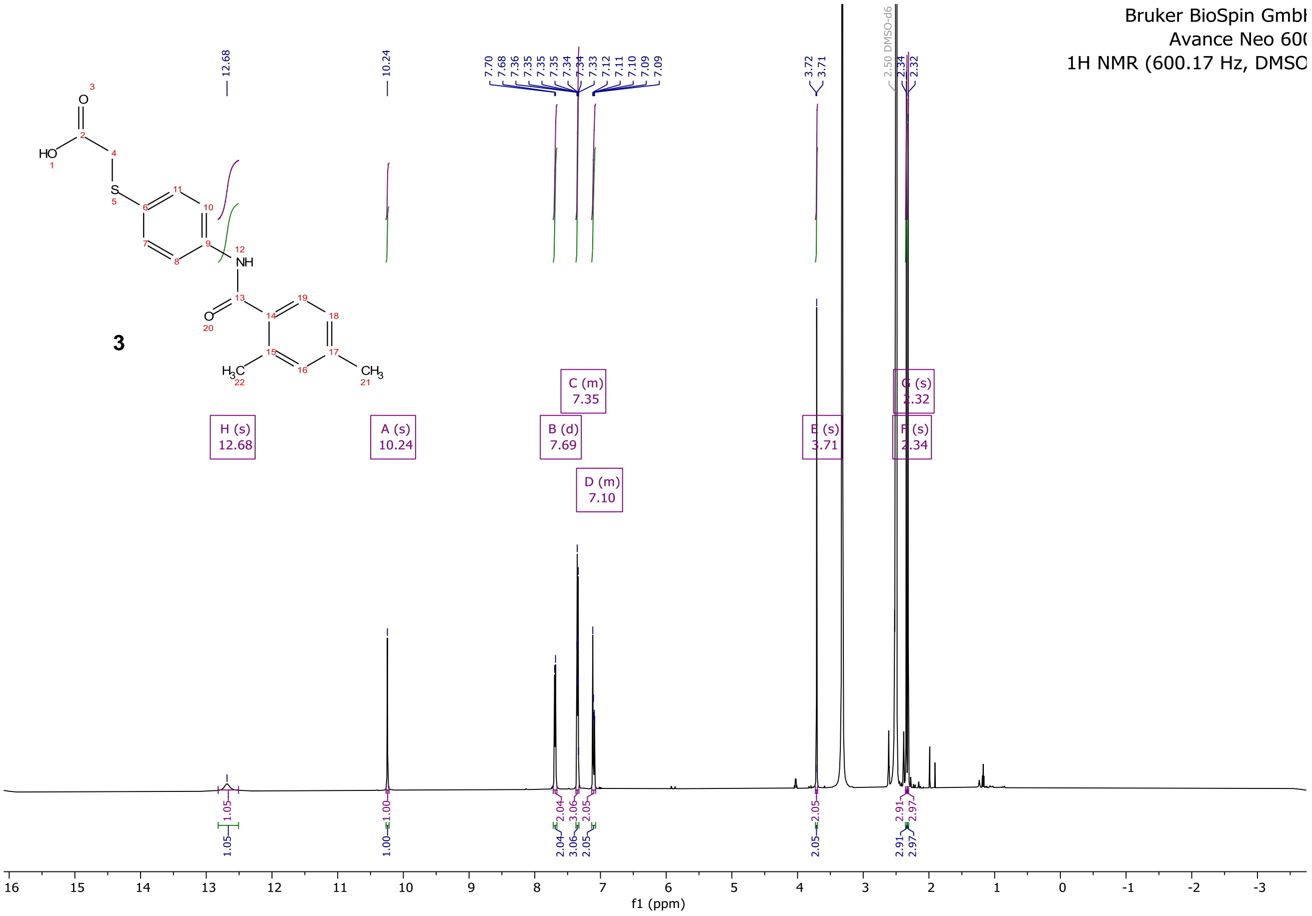

Bruker BioSpin GmbI
Avance Neo 60(
1H NMR (600.17 Hz, DMSC
3
H (s) 12.68
A (s) 10.24
B (d) 7.69
C (m) 7.35
D (m) 7.10
E (s) 3.71
F (s) 2.34
G (s) 2.32
2.50 DMSO-d6
f1 (ppm)

2,4-dimethyl-N-(4-((2-oxo-2-((4-phenylthiazol-2-yl)amino)ethyl)thio)phenyl)benzamide, F7H-28

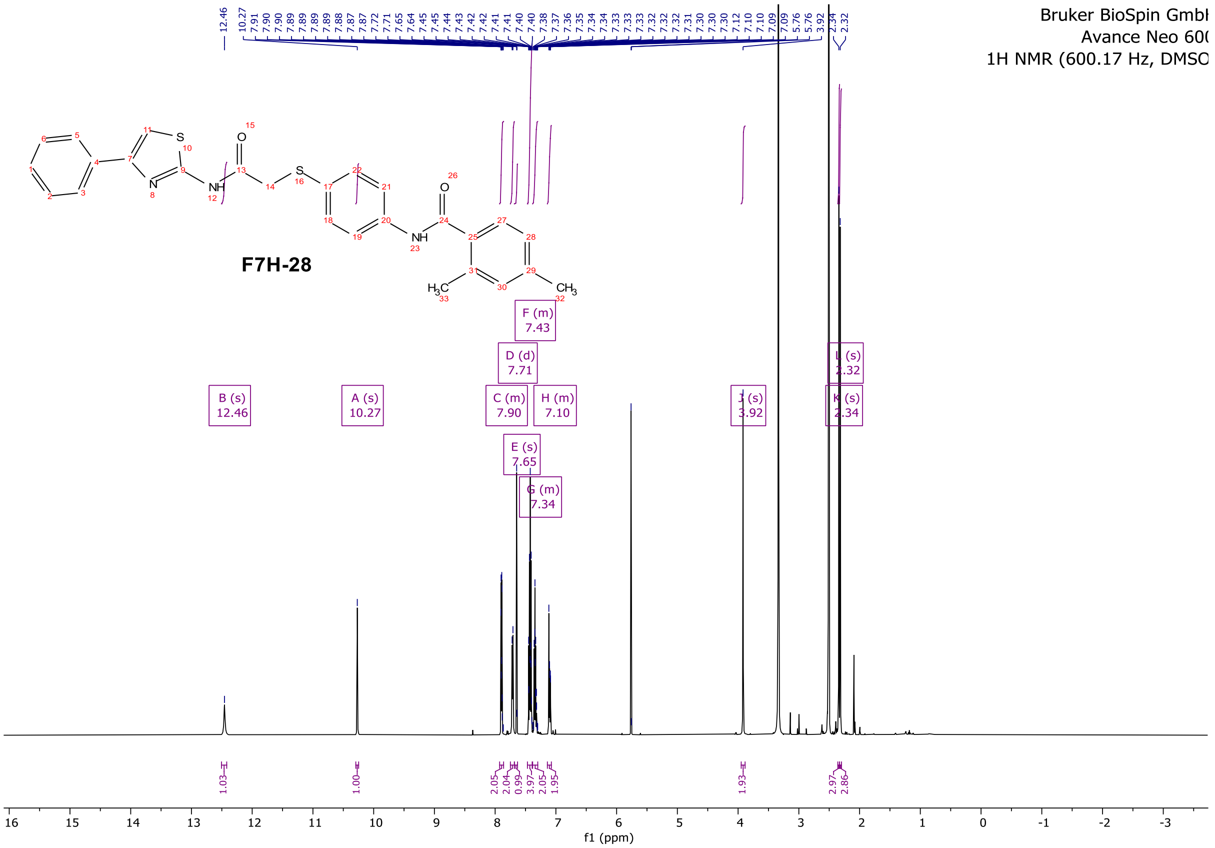

CBO-005-030-04.11.fi
13C NMR (150.93 Hz, DMSO

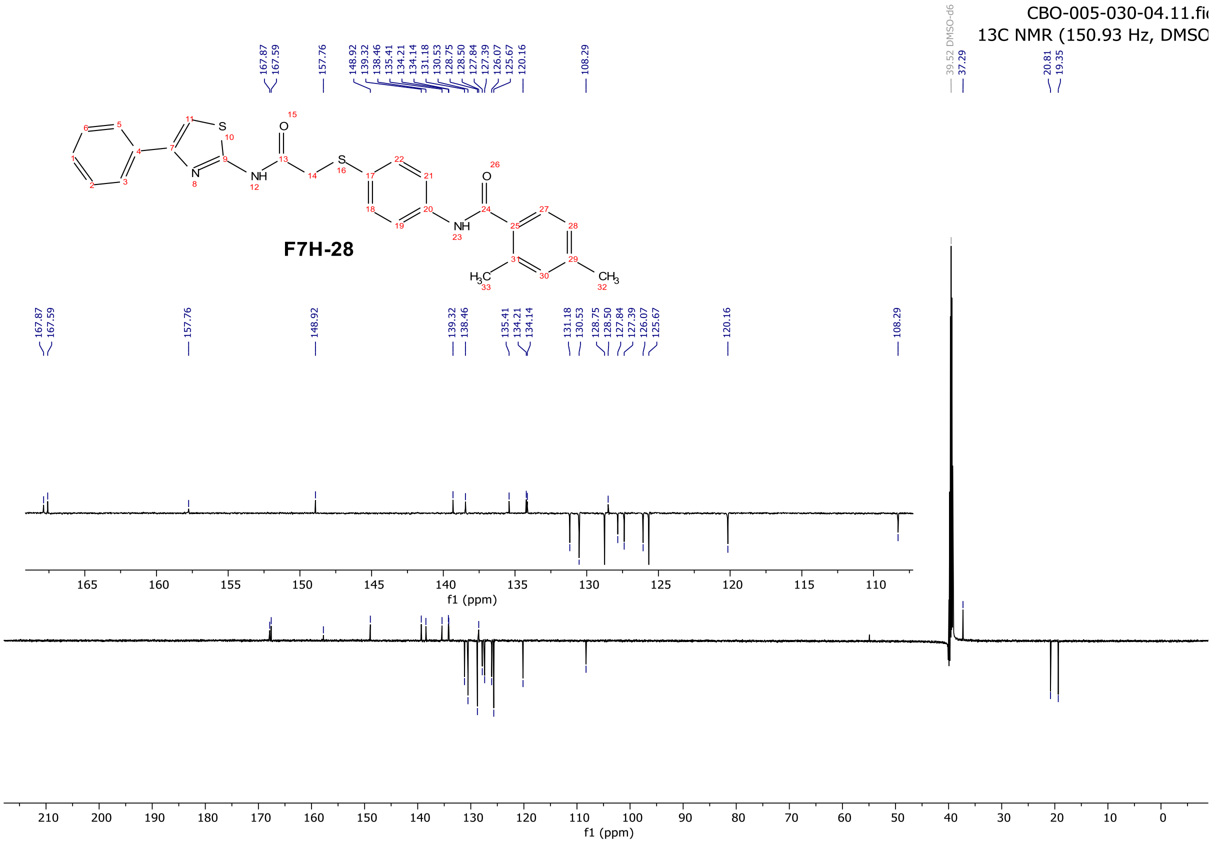

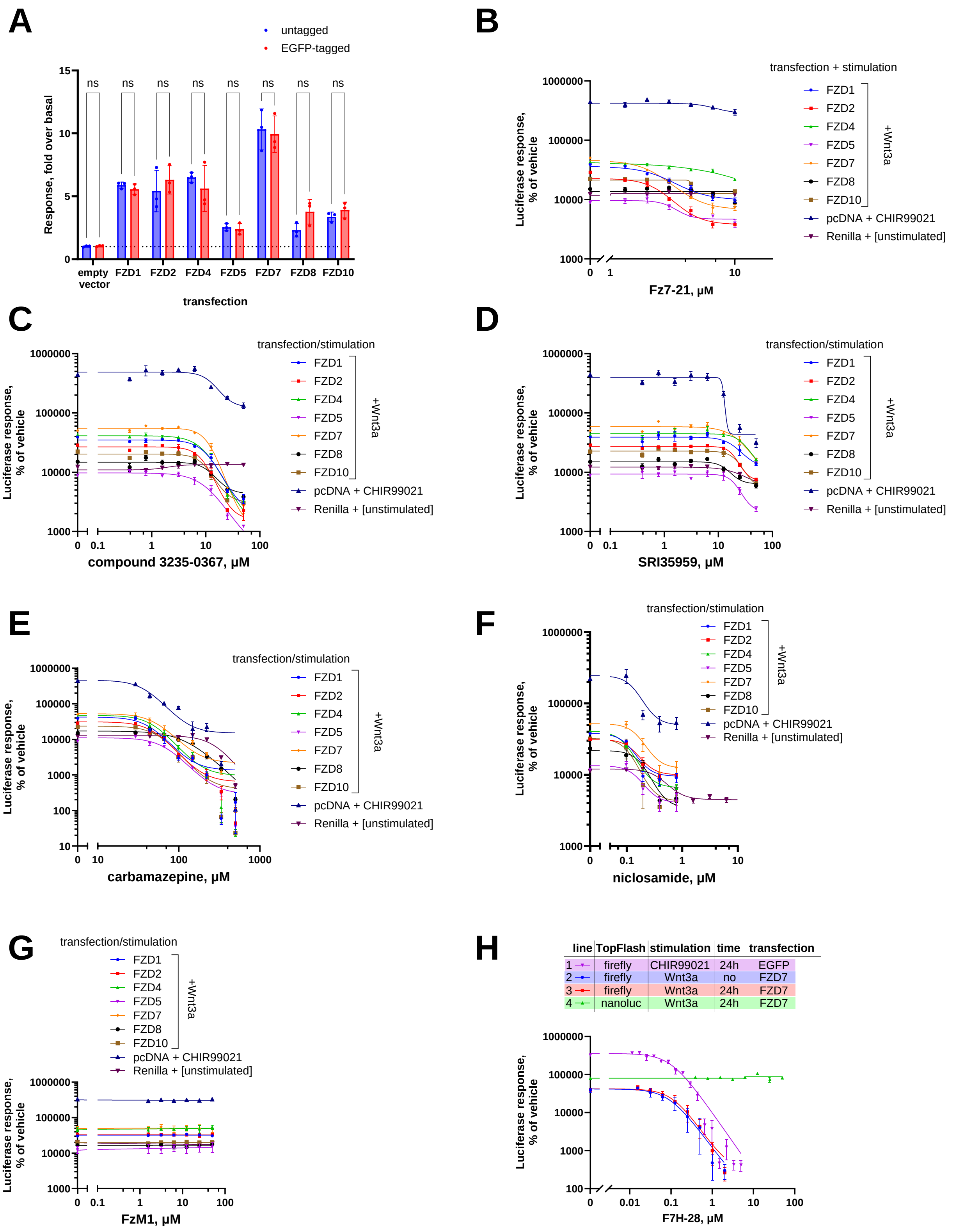

Figure S1: (A) Comparison of the relative response of the untagged and N-terminally EGFP-tagged constructs to Wnt3a in the ΔFZD1-10 HEK293 line after plasmid retransfection. **(B-H)** The graphs of raw counts corresponding to the normalized graphs in Figures 2-5.
